# A day in the life of third-year medical students: using an ethnographic method to understand information seeking and use*

**DOI:** 10.5195/jmla.2017.95

**Published:** 2017-01

**Authors:** Andrea B. Twiss-Brooks, Ricardo Andrade, Michelle B. Bass, Barbara Kern, Jonna Peterson, Debra A. Werner

## Abstract

**Objective:**

The authors undertook this project to learn how third-year medical students seek and use information in the course of daily activities, especially activities conducted in clinical settings in a variety of institutions.

**Methods:**

We recruited sixty-eight third-year undergraduate medical school students to create a mapping diary of a day that included clinical activities. We conducted semi-structured interviews based on the mapping diaries. Using content and thematic analyses of the resulting interview transcripts, we developed an ethnographic case study for each participant.

**Results:**

In the studied sample, we identified a broad range of information resources used for personal, clinical, and educational use. Participants relied heavily on technology throughout their day, including desktop computers, smart phones, handheld tablets, and laptops. Time management was a pervasive theme in the interviews, with participants squeezing in time to study for exams wherever and whenever they could. Selection of a particular information resource or technology to use was governed largely by the convenience of using that resource or technology. When obstacles were encountered, workarounds might be sought, but in many cases, the resource or technology would be abandoned in favor of a more convenient solution. Convenience was also a consideration in choosing spaces to use for clinical duties or for study, with specific considerations of available technology, proximity to clinical areas, and security for belongings contributing to choices made.

**Conclusions:**

Some of our results align with those of other recent studies of information use among medical students, residents, and practicing physicians. In particular, the fast-paced clinical setting favors use of information resources that are fast and easy to use. We demonstrated that the methods used are suitable to better understand clinicians’ discovery and use of information.

## INTRODUCTION

Applying ethnographic methods to understand information seeking and use can provide richer context about how and why users interact with information resources rather than other methods. For example, one might be able to use a survey to determine how prevalent use of a particular resource is, but not why a user chose that resource in the first place. While results are less generalizable than those from studies using other methods because ethnographic studies focus on specific incidences of information seeking and use, these studies produce a wealth of detail and description missing from surveys and similar methods.

Only a few studies have applied ethnographic techniques to study information seeking and use by medical students, residents, or physicians. In one recent mixed-method study, physicians and medical students recorded their movements in a diary for one week [1]. This was followed by semi-structured interviews that focused on factors underlying the use of a variety of electronic, text, or Internet resources. Reasons for use by students included checking for specific clinical information and learning purposes. Factors influencing choice of resource included speed and ease of access as well as quality of information. Reddy and Dourish used direct observation of staff in a surgical intensive care unit of a teaching hospital for several months combined with formal and informal interviews [2], finding that information seeking was seamlessly integrated into the workflow of the medical staff in that setting.

Other relevant non-ethnographic studies of medical school students included data collected by surveys, focus groups, and interviews. Factors influencing participants’ choice of information resources in these studies included ease of use, available time, authoritativeness, and barriers to use. In one survey of medical students, participants relied on point-of-care and other medical information electronic tools and on mobile device apps and search engines for quick fact-checking [3]. Students in a different study used generic search engines interchangeably with more specialized medical information resources for fact-checking and relied on subscription-based resources for more in-depth research questions [1]. In both studies, a login requirement was a deterrent, and participants sought alternative resources that did not require a password. One study found that when asked to read about their patients to prepare for rounds, students often turned to a clinical decision support tool [4], while in a different study, textbooks were preferred for this task [5]. Students sometimes avoided accessing information resources during patient encounters to not expose their lack of knowledge to patients or more senior physicians [1]. They also worried that using a mobile phone during patient encounters might be viewed as inappropriate [5]. Question banks or other electronic resources were generally preferred for studying for exams [6]. Medical residents’ use of information resources in clinical training environments was not dissimilar to that of medical students: they also identified speed, convenience, and access as important factors in selecting information resources [7, 8].

By employing an applied ethnographic method to investigate motivations and information seeking and use behaviors of third-year medical students in the authors’ various institutions, the authors aimed to develop a deeper and more nuanced understanding of the role of information use in clinical environments. We also hoped to use the results of this study to help inform decisions about library resources and services that support third-year medical students.

## METHODS

The target study population was third-year undergraduate medical students engaged in clinical clerkships at six Illinois medical schools. The institutions are a mix of private and public schools in urban and suburban settings. Participants constituted a sample of convenience at each institution. Table 1 provides a summary of the available demographic information.

**Table 1:** Participant demographics

Clerkship type	Count (n=68)
Anesthesia	2
Dermatology	1
Emergency medicine	1
Family medicine	6
Geriatrics	1
Internal medicine	8
Neurology	3
Obstetrics/gynecology	16
Pathology	1
Pediatric surgery	1
Pediatrics	6
Psychiatry	8
Surgery	9
Not specified	5

A study protocol was developed and agreed upon by the entire multi-institutional team and was based on a previously described mapping diary technique [9]. Individual institutional teams were responsible for obtaining institutional review board approval, recruiting participants from their own medical schools, obtaining signed informed consent forms, providing incentives for participation, and scheduling and completing interviews.

Ethnographic case studies were developed for each participant [10–12]. The approach employed a mapping diary, a paper map or log on which participants noted arrival and departure times for each location throughout a single day [13]. On the following day, participants completed a semi-structured interview in which they described their activities using the annotated map or completed log as a prompt [14, 15]. A sample map (supplemental Appendix A) and the protocol for the semi-structured interviews (supplemental Appendix B) are provided.

A total of sixty-eight interviews were conducted, audio-recorded, and transcribed. A full set of transcripts was provided to each institutional team for coding and further analysis. After a set of codes was developed by consensus, we coded the transcripts. Preliminary themes arose from the initial coding of the print transcripts, prompting additional rounds of coding (supplemental Appendix C). To facilitate manipulation and sharing of the data, we input coded transcripts into qualitative analysis software [16]. Content analysis of the coded transcripts was used to find specific mentions of information resources, technologies, and spaces utilized by students [10]. Thematic analysis of the transcripts identified topics of interest, including patterns of convenience in and obstacles to accessing information [11].

## RESULTS

Participants exhibited limited patterns of movement, but the maps proved valuable as a prompt for recalling detail during the interviews. Analysis revealed rich data about all aspects of “a day in the life” of these students, from the personal to the professional. Participants described their decision-making processes for selecting a particular resource. Decisions were based both on preferences for particular resources or features and on obstacles encountered in accessing and using resources. They made use of communication technologies, devices, social media, entertainment resources, educational materials, and clinical resources. Time management was a thread that ran throughout all the interviews, with participants juggling clinical duties, studying for exams, and carving out personal time. Other data in the transcripts provided insights into their understanding of professional conduct (or “putting on the white coat”).

### Information resources

Participants consulted a variety of information resources for clinically relevant information during the course of the study day. The most commonly mentioned resources are shown in Table 2. Information was used for personal learning (e.g., preparing for presentations, following up on a clinical issue, etc.), studying for exams, identifying literature for research projects, and providing patient care. Specific uses included finding drug dosing information and diagnostic criteria, searching for practice guidelines, exploring therapeutic options, reviewing videos of surgical procedures, finding images to aid in the interpretation of magnetic resonance imaging or computed tomography results, and checking standard values for lab results. Over fifty different clinical support tools, databases, books, study aids, and websites were named, and over two dozen different book titles (mainly print, but also e-books) were mentioned. Study guides were among the most used books (e.g., *Case Files, First Aid, Step Up to Medicine*), but more clinically oriented titles were also named (e.g., *Atlas of Pelvic Surgery, Harrison’s, Current Diagnosis & Treatment*).

**Table 2:** Occurrence of information resource by number of transcripts in which the resource is named

Resource	Number of transcripts (n=68)
UpToDate	51
Electronic health record (EHR)	53
Question review books or question banks	41
Google	35
Wikipedia	24
PubMed	23
Epocrates	16
Library website or page	15
Medscape	11

Personal uses of information included playing games, watching television programs, listening to radio programs, listening to music, reading news online, finding transportation information or using online maps and global positioning system (GPS) data, and using calendars. Social connections were ubiquitous, with participants describing activities such as texting, reading email, and checking social media (e.g., Instagram or Facebook) throughout the day, often at mealtimes or during breaks:

“I’ll check my email on my phone pretty frequently throughout the day, but really I don’t actually write back or spend much time with it unless it’s in the morning or at lunch.”

Social media was sometimes used for clinical and educational activities (e.g., checking a Facebook group set up for a student cohort or using texting to communicate with a resident on the team).

### Technology

Participants used a variety of technologies throughout the day for personal activities, clinical encounters, and studying. Smart phones were mentioned most often (in 62 or 91% of interviews), followed by desktop computers (55, 81%), laptop computers (43, 63%), and tablet devices (26, 38%). While pagers are still issued, they were mentioned by only 3 participants. Clinical and educational electronic resources were accessed using all these technologies, with many participants reporting the use of apps as well as standard web browsers on various devices.

Smart phones were often used during commuting times for directions, public transit arrival times, listening to music, doing practice questions, and studying. Throughout the day, all available technologies were used to check email, either personal or work-related accounts.

Of the students who participated in the study, only six explicitly mentioned using a mobile device with an Android operating system, while iOS smart phones and tablets were mentioned much more frequently. Occasionally, Android users reported running into barriers that iOS device users did not encounter:

“the only obstacle to actually using it [an mobile app] is that my tablet runs Android.”

The majority of students used a laptop for personal, professional, and educational computing needs away from their clinic or hospital sites. Multiple technologies were sometimes used simultaneously to aid studying, with the computer serving as the primary interface for exam question databases or applications and a tablet used for reference searching on the Internet (or vice versa).

### Spaces

The participants used a variety of spaces throughout the day, applying different selection factors as the situation dictated. Noise levels, proximity to clerkship location, availability of computers, strength of cellular or wireless access, and ability to interact with others were all mentioned as factors in selecting where they chose to work.

Students selected spaces to study in or to prepare to see patients that were close to their clerkship locations, such as physician or resident workrooms, the hospital cafeteria, or a nearby coffee shop. One participant admitted to selecting a café that he considered too expensive because it was convenient. Being around others, fellow students or the clinical team, was a factor:

“I prefer to be at a computer in the workroom with the residents…if something’s happening.”

The availability of computers often dictated students’ location choice. Not all hospital computers offered the same set of resources. Some computers had access only to electronic health records (EHRs) with no browsers; others had browsers that blocked certain sites. Students also chose locations in medical school spaces and libraries equipped with computers because they chose not bring their laptops with them on that day, either due to lack of security for their belongings or because they did not want to carry the extra equipment with them.

Certain spaces were considered uncomfortable or unconducive to studying due to factors such as the room temperature, noise, or amount of available space. Poor wireless or cellular network connections on the clinical floors were other obstacles encountered. When possible, students moved to other locations with stronger signals. Clinical spaces sometimes lacked a convenient, secure space for their belongings. In one case, a locker went unused because it was too far from the clinical setting. Belongings, including tablets and cell phones, were left unsecured in the workroom. Occasionally, students would leave their laptops or tablets at home due to concerns about the security of their belongings, even when having their devices would have been useful:

“but when we’re at the hospital, there’s not really a good spot to do that [store bags, coats, etc.], so I either leave the stuff in my truck or I leave it at home.”

## DISCUSSION

Results from this study echo many findings from previous studies. Participants made multiple decisions in their selection and use of information resources, technology, and spaces. Speed and convenience were important to users. Other factors—such as perceptions of behaviors, intended use for the information, or obstacles or barriers to use—affected decisions about selection and use of information resources, technology, and spaces.

### Quick answers during clinical encounters

Participants often had a very short time to find information for the clinical question of the moment. Resources that could quickly be accessed and searched were preferred to those that required a login and password. Likewise, those available in a locally downloaded app or one that stored login and password information were popular (e.g., Epocrates over Micromedex):

“[iPad] remembers my sign-ins and my log-ins.”

Google and Wikipedia were described as quick and convenient. Desktops with preconfigured portals and browsers with bookmarks for commonly consulted sites also provided quick access. Clinical sites that provided streamlined access to commonly used resources (e.g., EHR or UpToDate) were also preferred:

“Some hospitals will have subscriptions to it so that you can just click on the link on the desktop of any computer.”

This preference for “speed and ease” was similar to that found by other researchers [1]. In some clinical locations, significant problems with connectivity to wireless and/or cellular networks were noted. In one case, the student lacked a password to get on the wireless network; in another, the student walked to another different part of the building for a stronger signal. This was a source of considerable frustration:

“it’s kind of annoying because when you’re on the public wifi, when you move around or if you’re not logged in all the time it kicks you off.”

### Logical organization of information

UpToDate, Medscape, Google search results, and Wikipedia articles were popular for their well-organized presentation. Google was often a source for drug information; participants cited the well-formatted sidebar or “box” with summary information such as brand names, drug class, and side effects. Examination of examples of these boxes revealed that the information contained in them was often extracted from authoritative sources (e.g., www.FDA.gov).

### Recommendation from mentors and peers

Similar to findings from a previous study, the recommendation of an attending physician, a resident, or another student prompted the use of particular resources [1]. The participants also made use of resources that were provided and/or recommended by their medical schools, libraries, or clerkship programs.

### Format

Resources that use responsive design or are configured for easy perusal on a small screen were preferred when viewed on smart phones or tablets. In clinical sites where there were too few desktop computers or when students were moving around, this was an advantage. Participants also mentioned the ease of reading portable document format files (PDFs) on tablets. They valued small devices that fit in a pocket while they were in the clinical setting. One participant praised the iPad mini for this trait, while another noted that the regular iPad was too large to fit into scrub pants. Not all clinical settings were conducive to carrying a device in a pocket: one student noted that all devices were left outside of the labor and delivery room “just because it can get messy in there.” Several participants also mentioned using pocket-sized editions of print medical reference books:

“*Surgical Recall,* which is very helpful because it fits in my pocket.”

### Level of information need

When the participants required more than a quick answer to an immediate clinical question, they used resources that required more investment of time and effort. In preparing for presentations, studying for shelf exams, familiarizing themselves with upcoming procedures, or working on research projects, participants often used multiple information resources. In these situations, they were willing to deal with logins and passwords, pursue various alternatives to find full-text copies of articles, and try different search strategies to find more elusive information on topics.

### Other factors

Other factors contributed to decisions about use of particular resources. Remote access to EHRs and other resources was helpful to those working from home or from a clinical location outside their home campuses. Participants valued having different options or formats for information, sometimes employing one or more devices (e.g., a handheld tablet and a computer) along with a printed book. They expressed frustration when faced with a lack of an institutional subscription or license for needed journal articles. Traditional interlibrary loan services were deemed too slow to fill the gap in the cases when journal subscriptions were lacking:

“since I’m cutting the deadline a little short, I needed to have the full text last night.”

### “Putting on the white coat”

Social and professional factors also guided participants’ actions. In the clinical setting, they might be careful not to occupy computers that others perceived as higher ranking might need:

“but if the residents need the computers then the residents get the computers so when it gets crowded in there we get bumped down to the nursing station.”

The appropriateness of the use of smart phones was a frequent focus, similar to findings by Baudains [5]. Students almost always carried a smart phone with them, but whether to have it in vibrate or silent mode seemed dictated more by perceived professional conventions than individual student preferences. Some students were uncomfortable using their phones at all during clinical encounters, even to check on diagnoses and/or unfamiliar terms:

“Well, some attendings, like, will encourage you to use your phone because they also do, and it’s like, there are apps you know, that you can use to get information quickly. But the trouble with it is because if you just whip out your phone and start using it, then, and they don’t know what you’re trying to do, you look like you’re not paying attention, and that you’re, you know, on maybe doing social media or something like that. So I’m nervous to usually bring out my phone unless I say like, ‘Oh, here, I’ll look that up really quick on my phone,’ first.”

### Time management

The daily schedule of third-year students is extremely demanding, and many of their information, space, and technology use decisions were influenced by time management considerations. Smart phone alarm settings were utilized to ensure an on-time wake-up in the morning:

“I always double check. So I set [the alarms] then closed my phone and rechecked again…and then got up brushed my teeth, checked my alarm.”

Participants studied for shelf exams or prepared for other activities related to medical school, seemingly whenever they had a spare moment, wherever they happened to be. This could begin as they prepared for their day and often continued throughout the day: during their commutes, in the times between seeing patients, during lunch, and after they returned home:

“I didn’t do anything while I was waiting for the bus but once I was on the bus, I was using my phone to again do questions.”

“I got back in my car, checked Google maps on my phone…and listened [to an emergency medicine podcast].

Students used mealtimes and breaks, particularly lunch, for studying. Many students studied at home and often mentioned doing so right before going to sleep. Beyond that, students took advantage of increments of time to listen to lectures or read while commuting by bus, train, or car, and in one case, in an unexpected location:

“I love listening to lectures, listening to YouTube videos while I shower and while I get ready.”

## CONCLUSIONS

The libraries involved have used the data gathered during the study to make changes to services, spaces, and information resources to improve medical students’ experiences. One institution extended the borrowing period for iPads lent through the library to better accommodate the third-year students’ demanding schedules. The setups on computer workstations in another were changed to provide a more popular Internet browser. Yet another library changed the order of database resources listed on the portal page to reduce scrolling. At another institution, the availability of a mobile app for clinical resources was more aggressively promoted to students. The data were further used to guide a library renovation project, to improve a library teaching program, and to evaluate collection development approaches to e-books.

Data indicate that future actions by libraries should include strongly emphasizing responsive design for library resources to enhance usability on handheld devices, building a case for financial support for more types of clinical support and learning resources, and partnering with medical centers or schools to integrate more licensed information resources into the heavily accessed EHRs. More similarities than differences were noted among students from the six different institutions participating in this study, and it is expected that trends would likely be echoed at other institutions. Similar studies by other academic health sciences libraries could help build our knowledgebase for health sciences information use by our library communities. Such studies might focus on other clinical practitioner populations, dive deeper into specific aspects of information use, or build in other ways on this study.

## SUPPLEMENTAL FILES

Appendix A Sample annotated map showing limited movement

Appendix B “Day in the Life” mapping interview protocol form

Appendix C Sample codes used to aid transcript analysis
