# Supplementary material for: A day in the life of third-year medical students: using an ethnographic method to understand information seeking and use
Source: J Med Libr Assoc. 2017 Jan;105(1):12–9. doi: 10.5195/jmla.2017.95 (PMC5234461; doi:10.5195/jmla.2017.95)

JMLA Journal of the Medical Library Association

Supplemental content to
J Med Libr Assoc. Jan;105(1): dx.doi.org/10.5195/jmla.2017.95
www.mlanet.org
© Twiss-Brooks, Andrade, Bass, Kern, Peterson, Werner 2017

A day in the life of third-year medical students: using an ethnographic method to understand information seeking and use

Andrea B. Twiss-Brooks, MS, MLIS; Ricardo Andrade Jr., MLIS; Michelle B. Bass, PhD, MSI; Barbara Kern, MLIS; Jonna Peterson, MLIS; Debra A. Werner, MLIS

APPENDIX A

Sample annotated map showing limited movement

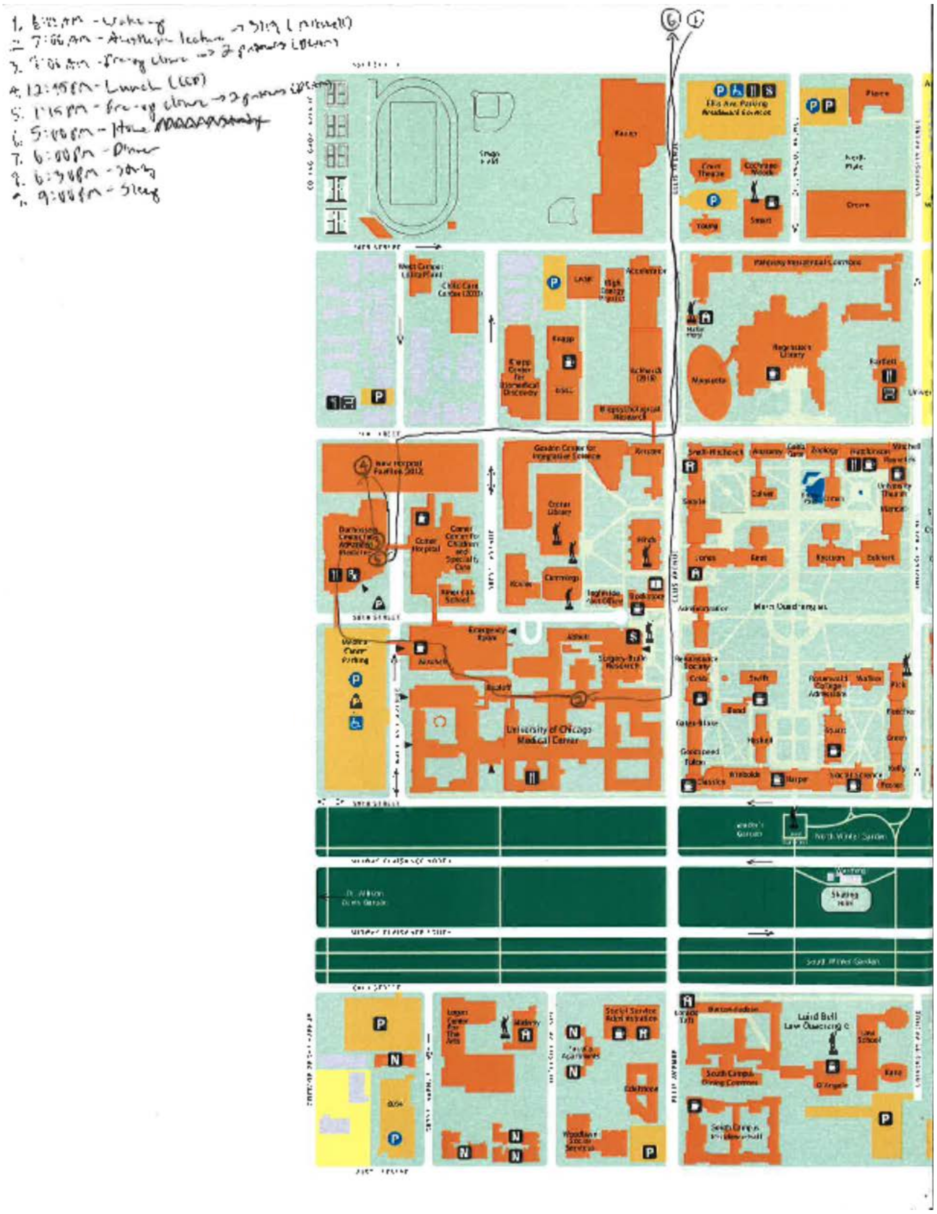

Supplement: Appendix A [file jmla_jan17_twiss_appendixa.pdf]
