# Supplementary material for: A day in the life of third-year medical students: using an ethnographic method to understand information seeking and use
Source: J Med Libr Assoc. 2017 Jan;105(1):12–9. doi: 10.5195/jmla.2017.95 (PMC5234461; doi:10.5195/jmla.2017.95)
Supplement: Appendix B [file jmla_jan17_twiss_appendixb.pdf]

JMLA Journal of the Medical Library Association

SC

Supplemental content to
J Med Libr Assoc. Jan;105(1): dx.doi.org/10.5195/jmla.2017.95
www.mlanet.org
© Twiss-Brooks, Andrade, Bass, Kern, Peterson, Werner 2017

A day in the life of third-year medical students: using an ethnographic method to understand information seeking and use

Andrea B. Twiss-Brooks, MS, MLIS; Ricardo Andrade Jr., MLIS; Michelle B. Bass, PhD, MSI; Barbara Kern, MLIS; Jonna Peterson, MLIS; Debra A. Werner, MLIS

APPENDIX B

"Day in the Life" mapping interview protocol form

Interviewee/participant code: ______________________________
Interviewer: ______________________________
[READ THE FOLLOWING AT THE BEGINNING OF THE INTERVIEW:]
For the transcript, I am going to read some prepared text before starting the interview.

This interview takes place at [INSTITUTION], conducted on ______________by
[DATE]
______________________ participant ______________________
[INTERVIEWER LAST NAME] [PARTICIPANT NUMBER]

When you were given your map to mark, you were also given information about the project and signed a consent form, including consent to have the interview recorded. Do you have any questions about the information on the form?

For your information, only researchers on the project will be privy to the recordings. These recordings will be destroyed after they are transcribed. Any personally identifying information will also be removed from the transcripts. In addition, all information from the study will be held confidential, your participation is voluntary, and you may stop at any time if you feel uncomfortable, and we do not intend to inflict any harm. Would you confirm now that you still agree to be audio recorded?

Thank you.

The research team has planned this interview to last no longer than thirty minutes.

You have been selected to participate in this project since you are a third-year medical student in [MEDICAL SCHOOL NAME]. The project focuses on information needs of medical students. The study does not aim to evaluate your techniques or experiences. Rather, we are trying to learn more about how medical school students discover and use information in the course of their activities, in clinical and other settings. We hope to use this information to help improve library services to better meet the needs of medical school students.

You have completed a mapping diary of your movements yesterday. I will be asking you to describe what you did at each location throughout the day. I may ask you to go into more detail about specific tasks or events. I am not trying to solicit any personal information that you might not be comfortable sharing, rather would like to get a sense of the structure of your day and how you find and use information throughout the day. Ready?
[STOP READING HERE AND PROCEED TO ASKING ABOUT THE MAP]

Journal of the Medical Library Association
Supplemental content to
J Med Libr Assoc. Jan;105(1): dx.doi.org/10.5195/jmla.2017.95
www.mlanet.org
© Twiss-Brooks, Andrade, Bass, Kern, Peterson, Werner 2017

Question guide

[DO NOT READ TO PARTICIPANT, THIS IS JUST AN EXAMPLE OF QUESTIONS:]

Interviewers will use the marked map from the mapping diary to ask the participants to describe their day and the activities and events of that day. Open-ended invitations to describe activities at each location will be used and may differ from participant to participant, depending on the specific events. The following are some examples of the types of questions that will be asked.

1. I see that you marked a place off the map where you started your day and that you spent ______ amount of time there. Tell me about your activities where and when you started your day.
2. How did you travel from where you started to your next location?
   a. [If participant used public transportation] Did you do anything to pass the time during your bus/train ride?
   b. Did you use a mobile phone or smart phone or some other device?
3. Once you arrived at your next destination, what did you do then?
4. [If participant mentions doing work for classes] Did you use the Internet for any of your class work? Tell me more about how you used the Internet, for example, use your own computer or some other workstation?
5. [If participant talks about use of information in a clinical setting] Without sharing any confidential personal health information of your patients, can you tell me about what kinds of information you looked for in support of your care of the patients? What kinds of resources did you use to find this information?

Background: mapping diary activity [to precede interview]:

Twelve third-year medical school student participants will receive a map of the campus and key surrounding areas and be asked to mark their movements on this map, indicating when they arrived at each place and when they left it. The resulting maps will be a record of how these individual students spent an actual day of their lives.

Maps will be full-color, eleven- by-seventeen-inch maps of campus on which they are asked to record the times and sequence of each event during a single day. After the students complete their maps, they will be interviewed by the principal investigator or one of the coinvestigators. The interviews will be audio-recorded and later transcribed.

Post interview comments and/or observations:

[USE THIS SPACE TO JOT DOWN ANYTHING THAT YOU MIGHT WANT TO FOLLOW UP ON BUT DON'T WANT TO DISRUPT THE INTERVIEW]
