# Supplementary material for: A day in the life of third-year medical students: using an ethnographic method to understand information seeking and use
Source: J Med Libr Assoc. 2017 Jan;105(1):12–9. doi: 10.5195/jmla.2017.95 (PMC5234461; doi:10.5195/jmla.2017.95)
Supplement: Appendix C [file jmla_jan17_twiss_appendixc.pdf]

JMLA Journal of the Medical Library Association

SC
Supplemental content to
J Med Libr Assoc. Jan;105(1): dx.doi.org/10.5195/jmla.2017.95
www.mlanet.org
© Twiss-Brooks, Andrade, Bass, Kern, Peterson, Werner 2017

A day in the life of third-year medical students: using an ethnographic method to understand information seeking and use

Andrea B. Twiss-Brooks, MS, MLIS; Ricardo Andrade Jr., MLIS; Michelle B. Bass, PhD, MSI; Barbara Kern, MLIS; Jonna Peterson, MLIS; Debra A. Werner, MLIS

APPENDIX C

Sample codes used to aid transcript analysis

Codes here represent the variety of codes used for content and thematic analyses of the transcripts.

Code name	Description or scope note
White coat	Use for any comments related to professional behavior/appearances, lore passed down from residents/attendings.
What information	Use for text where the medical information that they are seeking is explicated.
User interface	Use for any reference to searching, discoverability of resources/answers to questions, menu options, etc., especially with regard to applications used on the tablet.
Smart phone	Use for iPhone, Android Phone, Windows phone.
Discovery	Use to indicate electronic and other devices, print resources, online resources, communication tools, and other means of gathering information and to a lesser extent in disseminating information to others. Emphasis on medical information use, but nonmedical/leisure use is also coded.
Apps	Use in addition to codes that specify the type of resource used (e.g., Point of Care/UpToDate *and* apps would both be applied if specified in transcript).
Audio recordings or podcasts	Use for spoken word and music; if music instead of spoken word, also code "Leisure/nonmedical information."
Print books	For any print book. Individual titles are not coded separately but are indicated in a linked memo collected in the "Titles of Resources" memo group. If book is a study guide, also code in that category.
Study guide	Resources for exam preparation, either USMLE Step 2, Shelf Exams, or any other exams mentioned. May be print or e-book, code for format in addition to title when known.
Websites	Do not use for PubMed, Wikipedia, or Google, which have their own codes under Search Tools. Code any other websites using this code.
Devices	Code any electronic device use here, either for personal, educational, or professional use.
